# Local recurrence of breast cancer caused by core needle biopsy: Case report and review of the literature

**DOI:** 10.1016/j.ijscr.2020.06.013

**Published:** 2020-06-12

**Authors:** Kimiyasu Yoneyama, Motohito Nakagawa, Asuka Hara

**Affiliations:** Department of Breast Surgery, Hiratsuka City Hospital, 1-19-1 Minamihara, Hiratsuka-shi, Kanagawa 254-0065, Japan

**Keywords:** CNB, core needle biopsy, Breast cancer, Local recurrence, Core needle biopsy, Needle tract seeding

## Abstract

**Introduction:**

Needle tract seeding is the implantation of tumor cells at the site of needle passage during needle biopsy. Histopathological examination of resected specimens after biopsy shows an incidence of 22%–50%. However, reports of actual local recurrence are extremely rare. Here we report such a case.

**Presentation of case:**

A 67-year-old woman was diagnosed with ductal carcinoma by histopathology and underwent right mastectomy and sentinel lymph node biopsy. Histopathological examination revealed non-invasive ductal carcinoma. One year after the first operation, a mass was found at the site of the core needle biopsy (CNB) scar near the previous surgical wound on the right chest. Histological examination revealed the tumor as adenocarcinoma, and a skin lesion resection was performed. After surgery, radiation therapy and endocrine therapy were performed. She remains relapse-free as of this writing, 9 months after resection.

**Discussion:**

Reports of local recurrence due to needle tract seeding are extremely rare. We found nine cases of mastectomy and seven cases of partial resection performed for the first surgery; six patients received radiation therapy and 10 did not. Histological diagnosis at the time of the first operation was invasive carcinoma in all cases.

**Conclusion:**

The risk of seeding is high with multiple punctures in CNB, in cases with a short period until surgery, and in mucinous carcinoma. Considering these factors, CNB puncture should preferably be at a site that is included in the resection area during surgery. If not resected, close follow-up is necessary considering the possibility of local recurrence.

## Highlights

• This is the first report of noninvasive breast cancer (ductal carcinoma) recurrence caused by needle tract seeding.
• This type of recurrence is rare but preventable.

## 1 Introduction

Needle tract seeding is the implantation of tumor cells at the site of needle passage during needle biopsy. The incidence of needle tract seeding from histopathological examination of resected specimens after biopsy using 14–18G needles is reported to be as low as 22%–50% [1,2]. However, reports on actual local recurrences are extremely rare.

Here we report a case of breast cancer that recurred locally as a skin lesion, likely due to needle tract seeding 12 months after the first mastectomy for non-invasive ductal carcinoma.

This work was written in accordance with the SCARE criteria [3].

## 2 Presentation of case

A 67-year-old woman visited our hospital complaining of a right mammary mass.

**Fig. 1:**
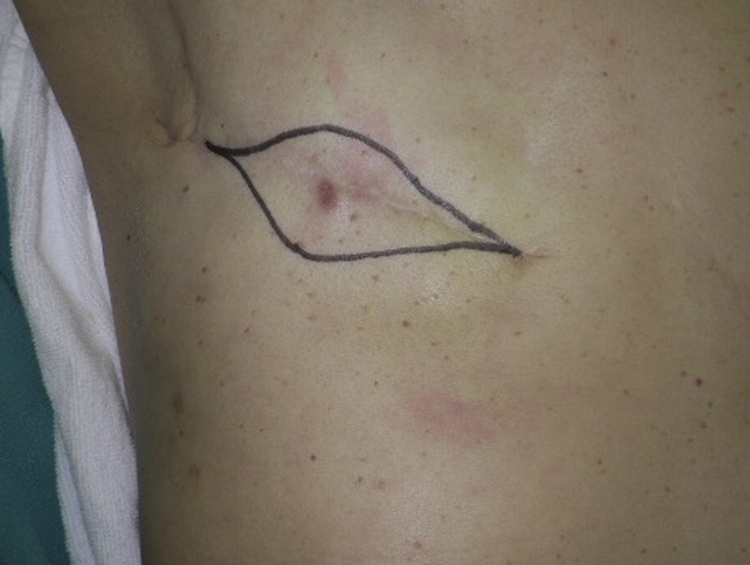
Skin mass in the same area as the CNB scar near the previous operation wound. Surgery involved extensive resection in a spindle-shaped manner.

**Fig. 2:**
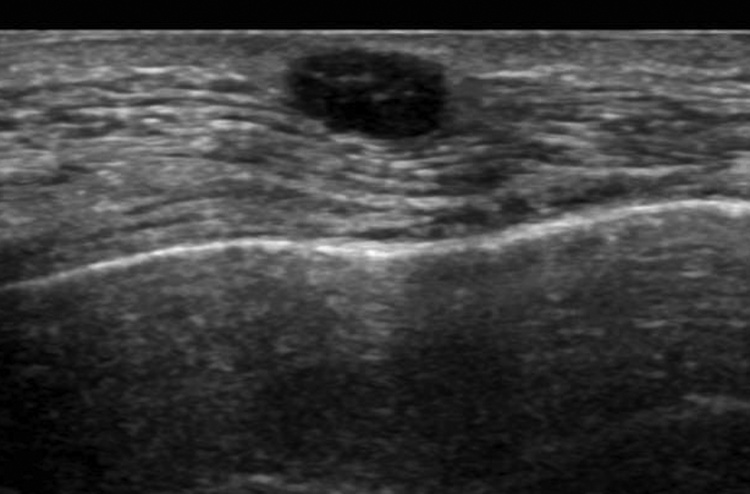
Ultrasonography showing a well-defined 8-mm diameter low-echo mass under the skin.

**Fig. 3:**
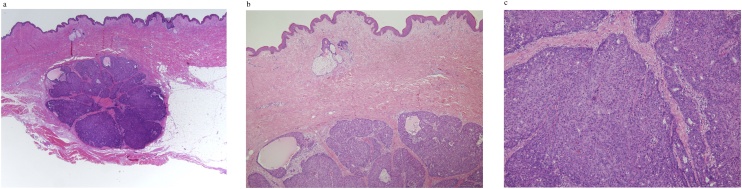
(a) The tumor is seen mainly in the subcutaneous tissue. No invasion into the pectoral muscle was observed. (b) Atypical cells with round, swollen nuclei and eosinophilic cytoplasm forming solid nests with palisade arrangement and gland duct formation.

Physical examination revealed a tumor 1 cm in diameter on the upper side of the right breast. Core needle biopsy (CNB) was performed due to suspected malignancy based on imaging, and ductal carcinoma was diagnosed based on histopathological examination. Right mastectomy and sentinel lymph node biopsy were performed. At mastectomy, the site of CNB scarring was not included for resection. The postoperative histopathological diagnosis was non-invasive ductal carcinoma, there was no lymph node metastasis, and the surgical margin was negative. No postoperative radiotherapy or endocrine therapy was administered, and only follow-up was conducted. There was a palpable skin mass 1 cm in diameter at the CNB scar site near the previous operation wound on the right chest on examination at 1 year after the first operation (Fig. 1). Ultrasound showed a tumor 8 mm in diameter in the subcutaneous tissue, suggesting skin metastasis (Fig. 2). Computed tomography scans showed no distant metastases, including to the lung, liver, and bone. Histopathological examination of a skin biopsy specimen revealed adenocarcinoma, and so a skin lesion resection was performed. Further histopathological examination revealed atypical cells with round, swollen nuclei and eosinophilic cytoplasm forming solid nests in the subcutaneous tissue. Palisade arrangement and gland duct formation were observed but there was no invasion into the pectoral muscles (Fig. 3a–c).

This was presumed to be a recurrence of a previous breast cancer, originating from the needle tract of the CNB. The resection margin was negative. Postoperatively, radiation therapy to the chest wall and oral administration of aromatase inhibitors were started. She remains relapse-free as of this writing, 9 months after resection.

The patient provided written informed consent prior to publication of this case report and the accompanying images.

## 3 Discussion

Needle biopsy has become an indispensable technique for diagnosis of various organ lesions and is more frequently performed than fine needle aspiration cytology in breast disease. A major advantage of needle biopsy is that it is relatively minimally invasive and a reliable diagnosis can be made, but one of the concerns is needle tract seeding. Needle tract seeding is the implantation of tumor cells at the site of needle passage during needle biopsy. Histopathologically, the needle tract is relatively easily observed as a linear distribution of mucous-like fibroblasts, granulation tissue, fat necrosis, cell debris, foreign body giant cells, and organized blood. The frequency of needle tract seeding on histopathological examination of resected specimens after biopsy using 14–18G needles reported is to be as low as 22%–50% [1,2].

Michaelopoulos et al. [4] reported that no malignant cells were found in the needle tract after vacuum-assisted breast biopsy with a 31G needle in 31 cases, and that no seeding of the needle tract was suspected if the treatment guidelines were followed. In addition, Robertson et al. [5] point out the difficulty of precisely distinguishing between true tumor cell dissemination and local recurrence, which may lead to underestimating the incidence of needle tract seeding.

In our case,

1) located far away from the primary tumor,
2) there is no mammary tissue around,
3) there is a lesion just below the CNB insertion site,

therefore, it was diagnosed as seeding by CNB.

Reports of local recurrence due to actual needle tract seeding are extremely rare, and as far as we can find, there are only 16 reports [[6], [7], [8], [9], [10]], making this the 17th.

Nine cases of mastectomy and seven cases of partial resection were performed for the first surgery. Six patients received radiation therapy and 10 patients did not. The time to recurrence ranged from 1 to 49 months with a median of 17 months. Histological diagnosis at the time of the first operation was invasive ductal carcinoma in 11 cases, mucinous carcinoma in 2 cases, and ‘other’ in 3 cases. All cases were invasive carcinomas.

To our knowledge, this is the first report of recurrence due to needle tract seeding in a case of non-invasive ductal carcinoma at the time of initial surgery.

Regarding the prognosis of local recurrence, in 9 of the described cases, median survival time was 29 (5–48) months, and no deaths were reported. Chao et al. [6] reported simultaneous or metachronous distant metastasis to other organs in breast cancer with skin recurrence with a poor prognosis. However, skin recurrence due to needle tract seeding does not fall into this category, and it is considered that the prognosis is not poor.

Liebens et al. [1] also stated that iatrogenic needle tract seeding after CNB did not increase morbidity.

Needle tract seeding does not always lead to clinical local recurrence for the following possible reasons:

1) The puncture scar is usually resected together with the lesion.
2) Needle tract seeding is controlled with adjuvant therapy, especially irradiation.
3) Needle tract seeding may be eliminated by host immune response or tumor cell apoptosis.

In our case, the CNB insertion site was away from the primary tumor and was not included in the resection range. Since the tissue was non-invasive ductal carcinoma, postoperative irradiation was not performed. Based on the above, it is speculated that a recurrence has occurred.

In pathological examination of resected specimens, seeding is often seen when the period from needle biopsy to surgery is short, and in 42% of the 352 cases reported by Diaz et al. [2] the interval from biopsy to resection was <15 days. Displacement was reduced to 31% between 15 and 28 days and to 15% for cases longer than 28 days.

Uematsu et al. [11] analyzed the surface of 18 G puncture needle using washing cytology.

Cells were positive in 134 out of 207 biopsies (65%), with a high rate of 77% in the multiple-puncture group compared with 66% in the single-puncture group. The positive rate for lobular carcinoma was 25%, which is significantly lower than that for other histological types.

The difference in histological type, which is low in lobular carcinoma and high in mucinous carcinoma, is interesting because intercellular adhesion is implicated in seeding.

Although there are few clinical cases where needle tract seeding progresses to overt cancer, efforts should be made to avoid local recurrence as much as possible.

To that end, the following should be considered.

1) Needle biopsy puncture should be performed at a site included in the resection range.
2) Puncture should be performed as few times as possible to prevent dissemination at the time of examination. The needle should be washed or replaced when performing multiple punctures. An outer cannula should be used if replacing the needle is difficult.
3) The needle tract should be resected at the time of surgery, especially in cases where there is a high possibility that postoperative radiation will not be performed.
4) Postoperative radiation should be considered if the needle tract cannot be removed.

After our experience with this case, we now try to remove the puncture site.

## 4 Conclusion

We encountered a case of local recurrence that was likely due to needle tract seeding at 12 months after radical surgery.

There is a high risk of seeding in cases with multiple punctures, in cases with a short time to surgery, and in mucinous carcinoma. Considering these factors, it is preferable to select a puncture site that can be included in the resection area during surgery.

If resection is not performed, close follow-up is necessary, taking into consideration the possibility of local recurrence from the same site.

## Declaration of Competing Interest

The authors declare that there is no conflict of interest regarding the publication of this article.

## Funding

Our study has not received any grant of funding.

## Ethical approval

Our institution does not require ethical approval for case reports that are deidentified and collected retrospectively.

## Consent

Written informed consent was obtained from the patient for publication of this case report and accompanying images.

## Author contribution

Kimiyau Yoneyama contributed to operation and writing the manuscript.

Asuka Hara contributed to operation.

Motohito Nakagawa reviewed the work.

## Registration of research studies

This is a case report, and no database approval was applied.

## Guarantor

Kimiyasu Yoneyama.

## Provenance and peer review

Not commissioned, externally peer-reviewed.

## CRediT authorship contribution statement

**Kimiyasu Yoneyama:** Conceptualization, Investigation, Resources, Writing - original draft, Writing - review & editing. **Motohito Nakagawa:** Supervision. **Asuka Hara:** Conceptualization, Investigation.

## References

[1] Liebens F., Carly B., Cusumano P., Van Beveren M., Beier B., Fastrez M. (2009). Breast cancer seeding associated with core needle biopsies: a systematic review. Maturitas.

[2] Diaz L.K., Wiley E.L., Venta L.A. (1999). Are malignant calls displaced by large-gauge needle core biopsy of the breast?. Am. J. Roentgenol..

[3] Agha R.A., Borrelli M.R., Farwana R., Koshy K., Fowler A., Orgill D.P. (2018). The SCARE 2018 statement: updating consensus Surgical CAse REport (SCARE) guidelines. Int. J. Surg..

[4] Michalopoulos N.V., Zagouri F., Sergentanis T.N., Pararas N., Koulocheri D., Nonni A. (2008). Needle tract seeding after vacuum-assisted breast biopsy. Acta Radiol..

[5] Robertson E.G., Baxter G. (2011). Tumour seeding following percutaneous needle biopsy: the real story!. Clin. Radiol..

[6] Chao C., Torosian M.H., Boraas M.C., Sigurdson E.R., Hoffman J.P., Eisenberg B.L. (2001). Local recurrence of breast cancer in the stereotactic core needle biopsy site: case reports and review of the literature. Breast J..

[7] Thurfjell M.G., Jansson T., Nordgren H., Bergh J., Lindgren A., Thurfjell E. (2000). Local breast cancer recurrence caused by mammographically guided punctures. Acta Radiol..

[8] Uriburu J.L., Vuoto H.D., Cogorno L., Isetta J.A., Candas G., Imach G.C. (2006). Local recurrence of breast cancer after skin-sparing mastectomy following core needle biopsy: case reports and review of the literature. Breast J..

[9] Kawasaki T., Ishida M., Tada T., Matsuya H., Saitoh M., Sato A. (2015). Well-differentiated neuroendocrine tumor of the breast with recurrence due to needle tract seeding. Virchows Arch..

[10] Hashimoto S., Oura S., Ohta F., Okamura Y. (2013). Two cases of breast cancer with skin recurrence due to needle tract seeding. J. Jpn. Surg. Assoc..

[11] Uematsu T., Kasami M. (2008). Risk of needle tract seeding of breast cancer: cytological results derived from core wash material. Breast Cancer Res. Treat..

